# Leukemic phase of ALK-negative anaplastic large cell lymphoma in a patient who is on androgenic steroids: A case report

**DOI:** 10.1016/j.amsu.2019.11.007

**Published:** 2019-11-22

**Authors:** Ganesh Kasinathan

**Affiliations:** Department of Hematology, Jalan Mewah Utara, Pandan Mewah, 68000, Ampang, Selangor, Malaysia

**Keywords:** *Hallmark cells*, *Horseshoe nuclei*, *Anaplastic*, *Lymphoma*, *Androgenic steroids*

## Abstract

ALK-negative anaplastic large cell lymphoma (ALCL) is a peripheral T-cell lymphoma that usually involves lymph nodes or extranodal sites. Leukemic phase of ALK-negative ALCL is exceedingly rare and often carries a poor prognosis. Androgenic steroids have gained popularity among the young, and at higher doses, it can result in immune dysregulation and may be potentially carcinogenic. Case presentation: A 30-year-old gentleman of Malay ethnicity presented to the hematology department with night fevers, loss of weight and bony pain for the past 6 weeks. He is a gymnasium instructor with a history of chronic usage of intramuscular testosterone enanthate. Physical examination revealed ecchymosis over the left elbow and hepatomegaly. A complete blood count depicted anemia, thrombocytopenia and leucocytosis. An 18-Fluorodeoxyglucose positron emission tomography (18-FDG PET/CT) imaging showed a hypermetabolic anterior mediastinal mass of 6.8 × 7.0 × 6.5 cm with diffuse hypermetabolism in the liver, spleen and axial skeleton. The bone marrow trephine and mediastinal tissue histology were consistent with leukemic ALK-negative ALCL. He was treated with CHOEP (cyclophosphamide, doxorubicin, vincristine, etoposide, prednisolone) induction chemotherapy in which he required intensive antibiotic and blood support. He progressed with worsening B symptoms and new diffuse lymphadenopathies suggesting rapid dissemination of the disease. He subsequently succumbed to multiorgan failure with disseminated intravascular coagulopathy at the intensive care unit. Conclusion: Leukemic phase ALK-negative ALCL often carries a complex karyotype and requires early intensive polychemotherapy. Use of anabolic steroids depletes the ability of defending lymphocytes to remove tumour producing cells.

## Highlights

• Clinicopathological correlation is imperative in the diagnosis of leukemic phase ALK-negative anaplastic large cell lymphoma.
• Leukemic phase ALK-negative anaplastic large cell lymphoma progresses rapidly if the diagnosis is delayed.
• Anabolic steroids compromises the immune system and depletes the ability of lymphocytes to remove tumour producing cells.

## 1 Introduction

ALK-negative anaplastic large cell lymphoma (ALCL) is a peripheral T-cell lymphoma that usually involves lymph nodes or extranodal sites and affects predominantly older adults. Systemic-type ALCL represents 2–3% of all non-Hodgkin lymphoma cases [1]. 15–50% of all systemic ALCLs are attributable to ALK-negative subtype [1]. ALCL is defined as proliferation of large atypical pleomorphic lymphoid cells, also known, as hallmark cells which contain horseshoe nuclei and often strongly expresses CD30 [2]. Morphologically, it is indistinguishable from ALK-positive ALCL. Leukemic phase of ALCL is very rare and usually carries a poor prognosis. Leukemic phase is most commonly reported in ALK-positive ALCL in children [3]. Anabolic androgenic steroids are artificial agents which function via the androgen receptors and have gained popularity among the general public and athletes. At higher doses, it can result in many undesirable effects such as liver malignancy, thrombotic events, immune dysregulation and is potentially carcinogenic [4]. This case-study describes an aggressive leukemic phase ALK-negative ALCL in a young male adult who has been on chronic use of androgenic steroids.

## 2 Case presentation

A 30-year-old gentleman of Malay ethnicity presented to the department of hematology with night fevers, loss of weight, poor appetite and bony pain for the past 6 weeks. He works as a gymnasium instructor and regularly self-injects (intramuscular) testosterone enanthate 750 mg fortnightly for the past three years. He is single, a non-smoker and a teetotaller. He has no other significant past medical or family history.

Physical examination revealed a medium built gentleman with stable vital parameters. He had ecchymosis over his left elbow with no palpable lymph nodes. His liver was palpable at 4 cm without other organomegaly. Other systems were unremarkable.

His complete blood count revealed bicytopenia with peripheral leucocytosis. The other laboratory parameters are tabulated in Table 1.

**Table 1:** Tabulation of laboratory parameters.

Laboratory parameters	Values (unit and normal range)
Hemoglobin	10.6 (13.5–16.5 g/dL)
Total White Cell Count	20.5 (4–12 × 10^9^/L)
Platelet	12 (150–400 × 10^9^/L)
Lactate Dehydrogenase (LDH)	6358 (90–180 U/L)
Alanine Aminotransferase	34 (0–40 U/L)
Creatinine	95 (40–100 μmol/L)
Erythrocyte Sedimentation Rate (ESR)	70 (0–20 mm/h)
Prothrombin Time (PT)	11.5 (9.5–13.5 s)
Partial Thromboplastin Time (PTT)	34 (27–38 s)
Serum free testosterone (taken 2 weeks from the last testosterone injection)	67 (47–244 pg/mL)
Immunoglobulin A (IgA)	0.5 (0.8–3.0 g/L)
Immunoglobulin G (IgG)	6.4 (6.0–16.0 g/L)
Immunoglobulin M (IgM)	0.9 (0.4–2.5 g/L)
Ebstein-Barr virus (EBV) serology	Not detected
Anti-HIV-1, 2	Not detected
Hepatitis BsAg	Not detected

**Fig. 1:**
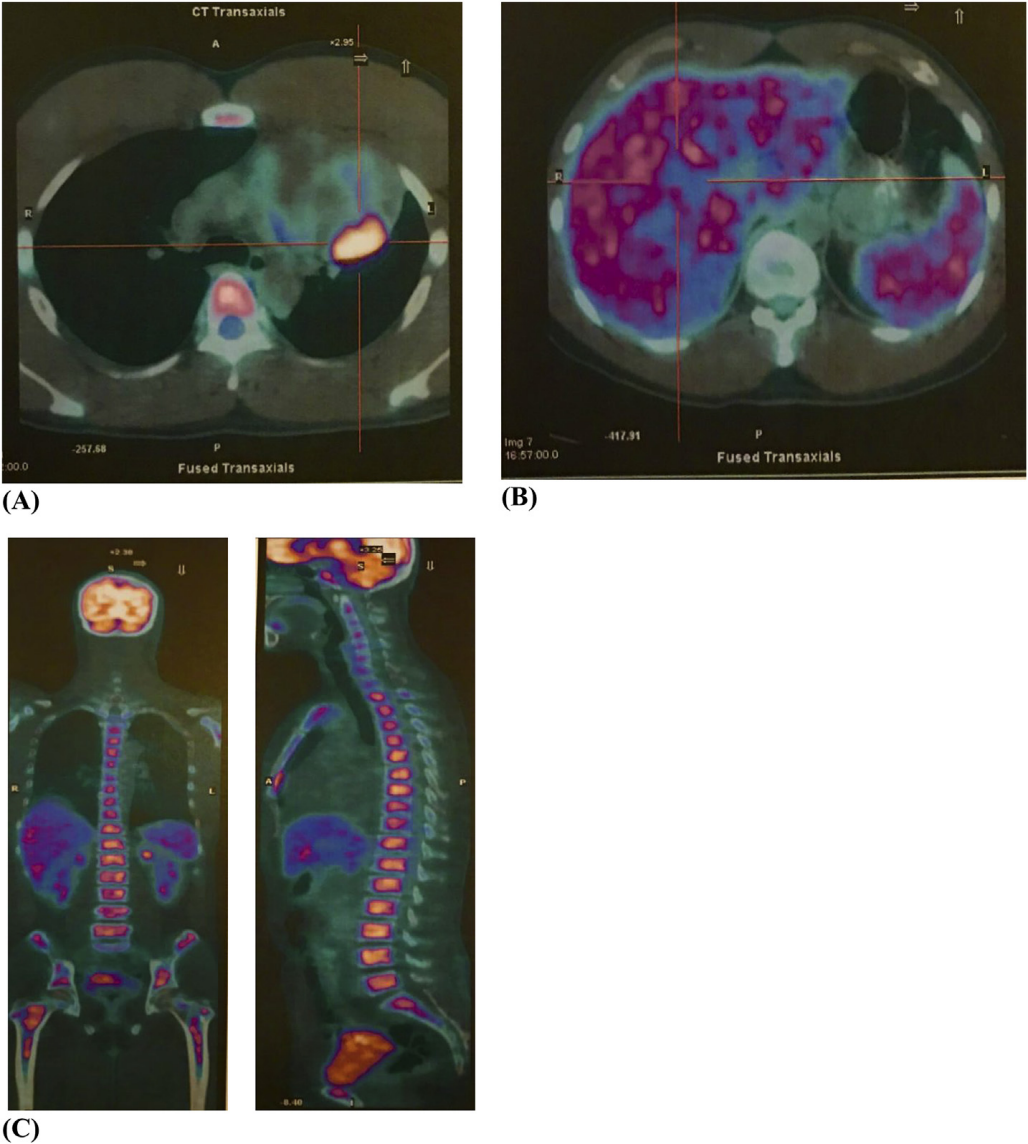
**(A, B, C):** 18- FDG PET CT whole body imaging. **(A).** The FDG imaging shows a well-defined 6.8 × 7.0 × 6.5 cm size and hypermetabolic left anterior mediastinal mass with a SUV (Standardised Uptake Volume) max: 9.5, Deauville 4. **(B):** Hepatomegaly present with a vertical span of 21.2 cm with a SUVmax: 5.9, Deauville 4 and the spleen demonstrates an SUVmax: 5.2, Deauville 4. **(C):** Diffuse hypermetabolic activity in the marrow of the axial skeleton, SUVmax:9.4, Deauville 4.

**Fig. 2:**
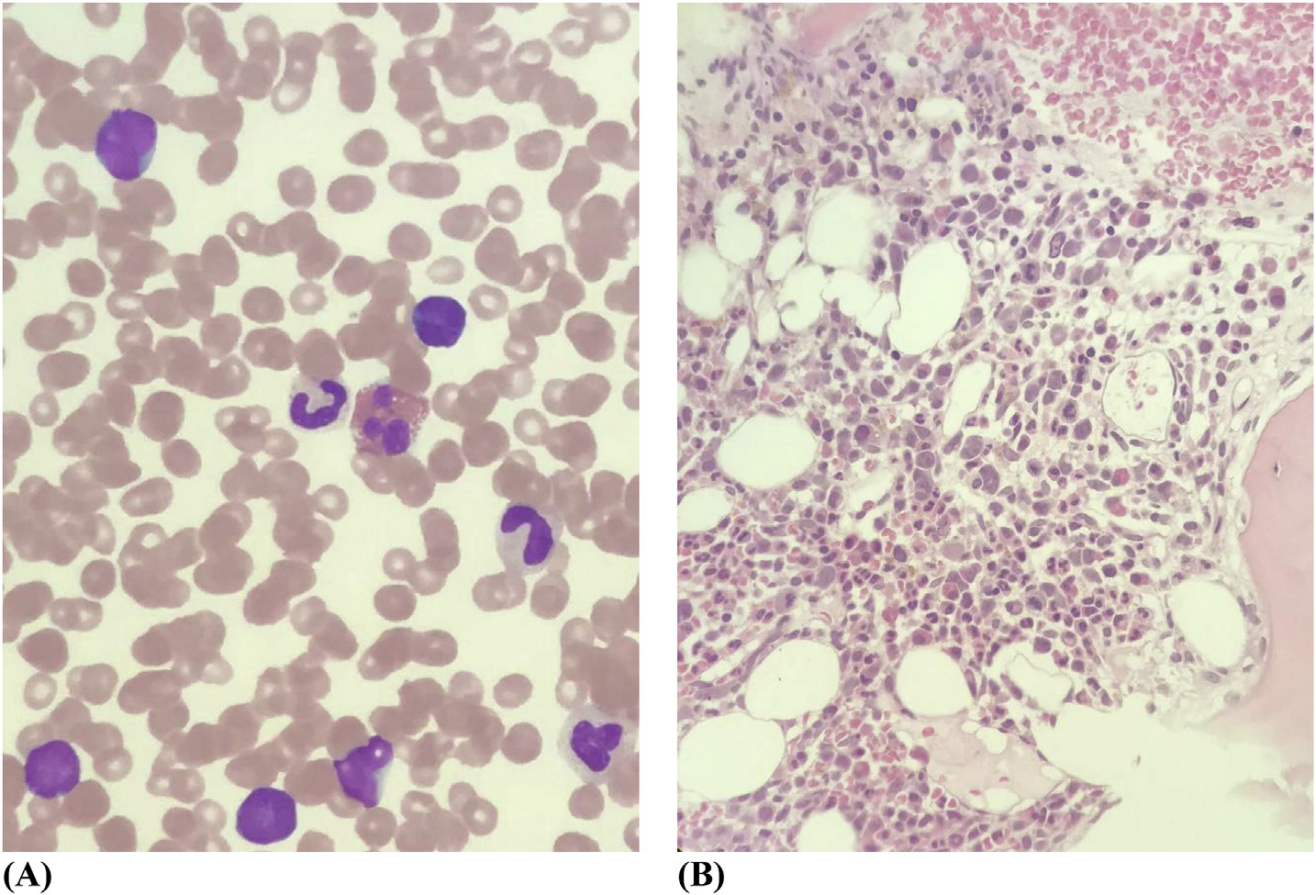
**(A)** Peripheral blood film shows abnormal lymphocytes. **(B)** The bone marrow trephine biopsy shows decreased granulopoiesis activity with diffuse replacement of marrow by large pleomorphic lymphoid cells and necrosis.

The peripheral blood film (Fig. 2A) showed 25% blasts, 55% abnormal lymphocytes, 12% neutrophils and 8% monocytes. The chest radiograph portrayed a widened mediastinum. The Whole Body 18-Fluorodeoxyglucose Positron Emission Tomography imaging (Fig. 1A, B & 1C) showed a hypermetabolic left anterior mediastinal mass of 6.8 × 7.0 × 6.5 cm with diffuse hypermetabolism in the liver, spleen and axial skeleton. Mediastinal tissue and bone marrow trephine histology (Fig. 2B) were consistent with ALK-negative ALCL. The malignant cells were positive for CD2, CD3, CD30 with MIB-1 activity seen in 60% of the cells. The cells were negative for Epstein-Barr virus-encoded small RNA 1 (EBER1), CD20, MUM1 and CKAE. A tissue microarray was constructed and the fluorescence in situ hybridisation (FISH) using chromosome break-apart probes for DUSP 22 and TP 63 loci were negative.

On the basis of the findings, a diagnosis of leukemic phase ALK-negative (triple negative) ALCL Stage IV B X age-adjusted International Prognostic Scoring index (aa-IPI: 4) was made.

He was given 2 cycles of CHOEP induction chemotherapy (cyclophosphamide 750 mg/m^2^, doxorubicin 50 mg/m^2^, vincristine 1.4 mg/m^2^, etoposide 100 mg/m^2^ and prednisolone 60 mg/m^2^) in 21-day intervals. He was compliant to chemotherapy. He required intensive antibiotic and blood support following the first CHOEP induction chemotherapy. His disease progressed on day 15 of the second CHOEP induction chemotherapy with worsening B symptoms, generalised bony pain with new cervical and inguinal lymphadenopathies suggesting rapid dissemination of the disease. He was scheduled for more intensive chemotherapy-methotrexate and cytarabine based polychemotherapy (Hyper-CVAD). However, he rapidly succumbed to multiorgan failure and disseminated intravascular coagulopathy at the intensive care unit.

## 3 Discussion

This is a rare case of a young adult male who presented with an aggressive ALK-negative ALCL which is consistent with the reported majority of patients who usually present with advanced stage, frequently manifesting B symptoms. Extranodal presentation with involvement of the skin, bone, lung and bone marrow is common. There are no particular risk factors associated with this disease. Those with an aggressive disease more often had a complex karyotype. Most cases of HIV and EBV-related ALCL are of B cell origin and are thus associated with Diffuse Large B Cell lymphoma-anaplastic variant [5]. There is an unclear role of Epstein Barr virus infection in T-cell or null-cell types of ALCL [5].

There are five morphological patterns of ALK-positive ALCL. They are common, lymphohistiocytic, small cell, Hodgkin-like and composite patterns. The tumour cells in ALK-negative ALCL demonstrate similar heterogeneity but the small cell pattern is not usually seen [6]. More than 80% of cases of ALCL in leukemic phase belong to the small cell pattern of ALK-positive ALCL [7]. However, this patient, belonged to the small cell group of ALK-negative ALCL which was in leukemic phase. The null-cell type of ALCL has loss of pan T-cell antigens. CD3 is negative in most ALK-positive ALCL whereas the CD2 and CD3 tend to show positivity in ALK-negative ALCL. The Epithelial membrane Antigen (EMA) is mostly positive in ALCL-positive ALCL but it is less common in ALK-negative ALCL [8].

Both subtypes of ALCL are dependent on IRF and MYC signalling. Next generation sequencing identified two recurrent rearrangements which carry prognostic value in ALK-negative ALCL which are DUSP22 and TP63 rearrangements. Those patients who harboured DUSP22 translocations had a better prognosis with a 5-year overall survival of 90% and were indistinguishable from ALK-positive ALCL. Patients with TP63 rearrangement had a much worse prognosis with a 5-year overall survival of 17% [9].

The use of anabolic androgenic steroids as illustrated in this patient contributes to dysfunction of the immune system. Several clinical studies reveal that they decrease antibody production, T and B lymphocyte stimulation and NK activity maturation resulting in immunosuppression [10]. Chronic steroid users had the lowest levels of immunoglobulins, particularly, IgA which could adversely affect the immune system as seen in this case. Furthermore, the cytotoxic activity of NK cells which is responsible to remove tumour producing cells is compromised [10].

CHOP (cyclophosphamide, doxorubicin, vincristine and prednisolone) chemotherapy remains the standard treatment for ALCL and good response is seen in ALK-positive ALCL except for those with multiple IPI adverse factors [11]. The NHB–B1 trial added etoposide to CHOP and shortened the therapy interval from 21 to 14 days (CHOEP-14) in young patients with aggressive ALCL [12]. This improved complete remission from 79% to 88% and 5-year event free survival by 12% [12]. Favourable outcomes have been demonstrated with ACVBP (doxorubicin, cyclophosphamide, vindesine, bleomycin, prednisolone) chemotherapy followed by consolidation therapy with methotrexate, ifosfamide, etoposide, asparaginase and cytarabine [12]. Addition of alemtuzumab which is an anti-CD52 monoclonal antibody to CHOP chemotherapy (Alemtuzumab-CHOP) for 4–8 cycles every 28 days has also shown good results [13].

After induction chemotherapy, high risk selected patients with ALK-negative ALCL may benefit from high dose therapy with BEAM (carmustine, etoposide, doxorubicin, melphalan) conditioning followed by autologous stem cell transplant in first remission [14].

Allogenic stem cell transplantation may be an option for relapsed ALCL in the younger group of patients. Transplant related mortality (TRM) is near 30% with myeloablative conditioning [14]. Reduced intensity conditioning (RIC) results in lower transplant related mortality and may have a role in certain circumstances [14].

Almost 50% of patients with systemic ALCL relapse after first line therapy. Relapse disease are usually resistant to conventional therapies. Brentuximab vedotin, an antibody-drug conjugate which targets CD30 is a promising agent in the relapsed setting as ALCL strongly expresses CD30 [15]. Follow up data has confirmed the safety and efficacy of brentuximab vedotin in treating relapsed/refractory systemic ALCL. Brentuximab vedotin in combination with cyclophosphamide, doxorubicin and prednisolone (CHP) as first line therapy in systemic ALCL is promising in Phase 3 clinical trials [15].

## 4 Conclusion

Leukemic phase ALK-negative ALCL is an aggressive malignancy which often has a complex karyotype and requires early intensive polychemotherapy. This disease progresses rapidly without appropriate therapy and may become life threatening if a patient delay seeking medical attention. Use of anabolic steroids may compromise the immune system, thus, depleting the ability of defending lymphocytes to remove tumour producing cells.

## Ethical approval

Not applicable this is not a clinical trial.

## Sources of funding

Self funding.

## Author contribution

The author contributed solely to the writing of this manuscript. GK wrote the first draft of the manuscript, contributed to the design, conception, structure and agreements for the paper. G.K made critical revisions and approved the final version of the paper.

G.K = Ganesh Kasinathan.

## Research registry number

Name of registry not applicable.

Unique identifying number: not applicable.

Hyperlink to the registration (must be publicly accessible) Not applicable.

## Guarantor

Ganesh Kasinathan is the guarantor of this manuscript.

## Declarations

Ethics approval and consent to participate: Not applicable as this is not a clinical trial.

## Consent

Written informed consent was obtained from the next of kin for publication of this case report and any accompanying images. A copy of the written consent is available for review by the Editor-in-Chief of this journal.

## Availability of data and material

Not applicable.

## Provenance and peer review

Not commissioned, externally peer reviewed.

## Declaration of competing interest

No conflicts of interest.
